# HIV-1 neutralization by monoclonal antibody against conserved region 2 and patterns of epitope exposure on the surface of native viruses

**DOI:** 10.1186/1476-8518-7-5

**Published:** 2009-10-12

**Authors:** Apichai Sreepian, Jongruk Permmongkol, Wannee Kantakamalakul, Sontana Siritantikorn, Nattaya Tanlieng, Ruengpung Sutthent

**Affiliations:** 1 Department of Microbiology, Faculty of Medicine Siriraj Hospital, Mahidol University, Bangkok, Thailand; 2 Faculty of Medical Technology, Mahidol University, Bangkok, Thailand

## Abstract

**Background:**

Conserved neutralizing epitopes are considered to be a key role for eliciting broadly neutralizing antibody (NAb). Previously, two conserved neutralizing epitopes of HIV-1 CRF01_AE envelope were identified at amino acid 93-112 of the C1 (C1E) and at 218-239 of the C2 (C2E) regions. To access the potency of antibody directed against conserved epitopes, a monoclonal antibody (MAb) specific to the C2E region was developed and characterized.

**Methods:**

The immunogenicity of two epitopes was examined by immunizing BALB/c mice with the matching synthetic peptides. One MAb, C2EB5, directed against peptide C2E, was generated by conventional methods, while C1E1 and C1E2 peptides induced slight antibody response in mice. The neutralizing activity of MAb C2EB5 was examined using a peripheral blood mononuclear cell (PBMC) based method and various HIV-1 subtypes including A, B, C, D, and CRF01_AE; C2EB5 was compared with other known neutralizing MAbs (4E10, 447-52D) and with sCD4. The exposure of the C2 epitope on native virus was investigated using virus capture by these MAbs.

**Results:**

The MAb C2EB5 demonstrated cross-neutralization against various HIV-1 subtypes. The overall potency of MAb C2EB5 against 5 subtypes was ranked in the following order: subtype C> CRF01_AE> subtype D> subtype A> subtype B. The epitope exposure for MAb C2EB5 was also correlated with the neutralization properties of each subtype.

**Conclusion:**

This study demonstrates the cross-clade neutralizing activity of a MAb directed against an epitope located in the C2 region of the HIV-1 env and highlights differences in the exposure of antigenic epitopes on the surface of various HIV-1 subtypes. The epitope for this newly identified neutralizing MAb made against a subtype CRF01_AE peptide is particularly exposed in subtype C viral isolates.

## Background

The great variability HIV-1 antigenic epitopes has been considered to be a major mechanism used by the virus to evade the host immune response. To elicit broadly neutralizing antibody (NAb) against HIV-1, one or more conserved epitopes should be recognized to overcome the extensive antigenic diversity. However, there are few conserved epitopes on the envelope protein that are accessible for specific antibody binding and neutralization. These epitopes have been hidden either by glycosylation or conformational masking [1,2]. The major targets of HIV-1 neutralizing antibodies are located on the surface gp120, whose diverse antigenic epitopes mediate receptor and co-receptor binding [3,4], and on the transmembrane gp41, which causes membrane fusion and allows the virus to gain entry into host cells [5]. A previous report has shown that one-third of neutralizing specificities of subtypes B and C neutralizing antibodies in polyclonal sera recognize the CD4 binding site (CD4b) and gp41 epitopes, while two-thirds of the antibodies were estimated to be directed against unidentified epitopes [6].

Three monoclonal antibodies (2G12, IgG1b12, 447-52D) directed against gp120 and three MAbs against gp41 (MAbs: 2F5, 4E10, Z13) have been extensively described in their neutralizing activities. Of the anti-gp120 MAbs, 2G12 recognizes a unique epitope in a carbohydrate-rich region on the outer domain involving the C3-V4 region [7,8], whereas IgG1b12 binds to the CD4 binding site and 447-52D recognizes the V3 loop of gp120 [9]. The anti-gp41 MAbs, 2F5, 4E10 and Z13 bind to the same continuous membrane proximal region of gp41. 2F5 is mapped to the conserved sequence ELDKWA [10], whereas 4E10 and Z13 recognize an epitope involving the sequence NWF(D/N)IT, which is located on the C-terminus of the 2F5 epitope [11,12]. There have been several MAbs developed against various conserved epitopes that show some neutralization, such as 17b and 48d. The MAbs 17b and 48d recognize a cluster of gp120 epitopes that are centered on the β 19 strand and partially overlap the co-receptor binding site [13,14]. While many of the known HIV Env MAbs are specific for conserved regions, several reports have demonstrated that some variable amino acid patterns lead to NAb resistance [15,16].

The emergence of circulating recombinant forms (CRFs) has been recognized and it is thought that they will make the HIV-1 epidemic more complex. This may have serious issues for the future of antiretroviral therapy and vaccine development. At least 32 circulating recombinant forms have been reported in HIV-1 group M [17]. CRF01_AE, a hybrid of subtype A (*gag*) and E (*env*), is an important HIV-1 recombinant form prevalent in Asia. Since we demonstrated some conserved neutralizable epitopes, which are located on amino acids 93-112 (C1 region) and 218-239 (C2 region) of HIV-1 CRF01_AE primary isolates in previous study [18], we have attempted to test the immunogenicity of these conserved epitopes and potencies of these induced MAbs. Toward that aim, we immunized BALB/c mice with peptides corresponding to these epitopes and MAbs specific to these epitopes were produced. A monoclonal antibody directed against peptide C2E (amino acids 218-239) was produced and the neutralization pattern for this C2EB5 MAb has revealed a cross-neutralizing activity and the presence of antigenic epitopes for this site on the surface of native viruses. The antigenic portion of this epitope appears to be particularly exposed in subtype C envelopes.

## Methods

### Monoclonal antibodies 4E10 and 447-52D and soluble CD4 (sCD4) [19]

Two human MAbs (4E10 and 447-52D) and sCD4 were kindly gifted from the National Institute for Biological Standards and Control (NIBSC, UK) whereas MAb C2EB5 was produced in this study [20]. The MAb 447-52D recognizes GPGR motif at amino acids 312-315 on the tip of V3 loop whereas MAb 4E10 recognizes NWFDIT located at amino acid position 671-676 in gp41. Soluble CD4 (sCD4) is an entry inhibitor devised as a decoy for the HIV-1 gp120 protein. These MAbs and sCD4 were aliquot and stored at -20°C.

### Primary isolates and T-cell line adapted (TCLA) strains of HIV-1

Five HIV-1 CRF01_AE primary isolates were obtained from National HIV Repository and Bioinformatic Center (Thailand) (NHRBC). These viruses with prefix MENO were collected from HIV-1 seropositive cases from the northern part of Thailand through National serosurveillance in the year 2002, including MENO12 (AY243187), MENO23 (AY243194), MENO24 (AY243195), MENO31 (AY243202) and MENO43 (AY243213). HIV-1 TCLA strains including, 2 subtype A (92RW009 and VI191), 4 subtype B (QH0692, SF162, IIIB and MN), 2 subtype C (92BR025 and DU174), 1 subtype D (92UG024) and 2 CRF01_AE (NP1525 and NPO3) were obtained from National Institute for Biological Standards and Control (NIBSC, UK). These viruses were thawed from liquid nitrogen and co-cultivated with PHA-stimulated donor PBMCs in IL-2 medium. The viral multiplications were followed up by measuring p24 level (Vironostika HIV-1 Antigen, bioMerieux). The value of 50% tissue culture infectious dose (TCID_50_) for each virus stock, both primary isolates and TCLA strains, was titrated on PHA-stimulated PBMCs and the value of TCID_50 _was calculated by Spearman-Karber method.

### Peptides corresponding to conserved neutralizable epitopes on C1 and C2 regions of gp120 (HIV-1 CRF01_AE)

The designation of peptides corresponding C1 (C1E1 and C1E2) and C2 regions (C2E) has been described previously [18]. The peptides were designed from alignment of *env *nucleotide sequences (C2-V4) obtained from 43 HIV-1 CRF01_AE primary isolates (GenBank under accession number AF373037-AF373043, AY005164-AY005179 and AF322195-AF322214) [18]. The amino acid sequence of peptide C1E1 (amino acids 93-112 of C1 region) is ENFNMWKNNMVEQMQEDVIS whereas amino acid sequence of peptide C1E2 is different from C1E1 at position 101, where an N residue is changed to a K, as underlined in Table 1. Peptide C2E, 22-mer peptide containing DPIPIHYCTPAGYAILKCNDKN, is located at residues 218-239 of the C2 region. The activities of these peptides have been investigated in previous studies by inhibiting the neutralizing activities of sera from long-term non-progressors (LTNPs) infected with HIV-1 CRF01_AE [18].

**Table 1:** Alignment of amino acid sequences of the Env glycoprotein gp120 at position 218 to 239.

	**Clade**	**Accession no.**	**218**	**219**	**220**	**221**	**222**	**223**	**224**	**225**	**226**	**227**	**228**	**229**	**230**	**231**	**232**	**233**	**234**	**235**	**236**	**237**	**238**	**239**	**240**
**C2E**			**D**	**E**	**P**	**I**	**P**	**I**	**H**	**Y**	**C**	**T**	**P**	**A**	**G**	**Y**	**A**	**I**	**L**	**K**	**C**	**N**	**D**	**K**	**N**
NP1525	AE	AAW57720	.	.	.	.	.	.	.	.	.	.	.	.	.	.	.	.	.	.	.	.	.	.	K
MENO23	AE	AY621208	.	.	.	.	.	.	.	.	.	.	.	.	.	.	.	.	.	.	.	.	.	.	.
MENO43	AE	AY621222	.	.	.	.	.	.	.	.	.	.	.	.	.	.	.	.	.	.	.	.	.	.	.
92BR025	C	AAB61124	.	.	.	.	.	.	.	.	.	A	.	.	.	.	.	.	.	.	.	.	N	.	T
DU174	C	DQ411853	.	.	.	.	.	.	.	.	.	A	.	.	.	.	.	.	.	.	.	.	N	N	K
SF162	B	P19550	E	.	.	.	.	.	.	.	.	A	.	.	.	F	.	.	.	.	.	.	.	.	K
QH0692	B	AY669730	F	.	.	.	.	.	.	.	.	A	.	.	.	F	.	.	.	.	.	.	N	.	T
IIIB	B	AB037858	E	.	.	.	.	.	.	.	.	A	.	.	.	F	.	.	.	.	.	.	N	.	T
MN	B	P05877	E	.	.	.	.	.	.	.	.	A	.	.	.	F	.	.	.	.	.	.	.	.	K
VI191	A	ABY26917	E	.	.	.	.	.	.	.	.	A	.	.	.	F	.	.	.	.	.	R	.	.	E
92RW009	A	AY669700	F	.	.	.	.	.	N	.	.	A	.	.	.	F	.	.	.	.	.	K	.	.	K
92UG024	D	AAT67532	E	.	.	.	.	.	.	.	.	A	.	.	.	F	.	.	.	.	.	.	N	.	M

Dots and dashes indicate similarity of amino acids, respectively.

### Immunization and monoclonal antibody production

Six to eight week old female BALB/c mice (from National Laboratory Animal Center, Thailand) were immunized with synthetic peptides by intraperitoneal injection protocols. Two groups of mice (2 mice/group) were primed with 100 μg peptide/100 μl complete Freund's adjuvant (Sigma, USA). Two weeks later, the first group was intraperitoneally boosted with 100 μg peptide/100 μl incomplete Freund's adjuvant (IFA) (Sigma, USA) whereas the second group was boosted with 200 μg peptide/100 μl IFA. For control groups, the mice were immunized with normal saline instead of peptide utilized the same preparation of peptide immunizations. All mice were bled and sacrificed after boosting 3 days, and then all sera was kept frozen. The spleenocytes were separated immediately to hybridize with myeloma cells Ag8.653 by using 41.3% polyethylene glycol (Sigma, USA) as fusion reagent [20]. The hybridoma cells were cultured in HAT medium, RPMI 1640 medium supplemented with hypoxanthine-aminopterin-thymidine (Sigma, USA) and 20% FBS, for a week before transferring to HT medium, medium without aminopterin, until the colonies of hybridoma cells were grown. Initially, the hybridoma cells were diluted in round-bottom 96-well plate by limiting dilutions to obtain 1-10 cells per well and cultured for 5-7 days. The supernatant from each well was screened for antibody by peptide ELISA to identify the antibody producing clones. Then, they were subcloned by limiting dilutions (< 1.0 cell per well) twice and antibody positive clones were selected. Monoclonal IgG was purified by passing culture supernatant through ProPur™ protein G spin column (Nunc, Denmark) according to the manufacturer's instruction. IgG purity was determined by SDS-PAGE and Western blot with goat anti-mouse conjugated with HRP (Invitrogen, USA).

### Detection of antibody responses in BALB/c mice and antibody produced in hybridoma cell lines by peptide ELISA

The flat-bottom 96-well plates were coated with 100 μl of peptide (5 μg/ml carbonate buffer, pH 9.6) for overnight at 4°C. Following washing steps, the plates were blocked with blocking buffer (5% skimmed milk, 0.3% Tween20 in PBS) for 1 h. The plates were washed again before incubating with 100 μl of sera from BALB/c mice (for detect Ab response in BALB/c mice) or 100 μl of culture supernatants (for detect Ab production in hybridoma cell culture) for 1 h at 37°C. After washing steps, 100 μl of HRP-conjugated goat-anti mouse IgG (Invitrogen, USA) was added and the plates were allowed to incubate for 1 h at 37°C. Then, 100 μl of TMB substrate (Zymed, USA) was added and the reaction was stopped with 100 μl of 1 M H_2_SO_4_. The absorbance was measured at wavelength 450 nm. The cutoff is defined as the mean value of absorption of serum samples from mice immunized with normal saline or that of fresh culture medium.

### HIV-1 neutralization assay

Neutralization test was assayed by a method based on PBMC infection and reduction of p24 gag protein in culture fluids, as described previously [18]. Briefly, 75 μl of virus supernatant (30 TCID_50_) was pre-incubated with equal volume of serially diluted MAb or sCD4 at 37°C for 1 h. After that, 75 μl of PHA-stimulated PBMCs (1.34 × 10^6 ^cells/ml) was added and allowed to incubate at 37°C, 5% CO_2 _for 18 h. The infected cells were then washed twice and re-suspended in 400 μl of IL-2 medium before transferring 200 μl of cell suspension into duplicated well of round-bottom 96-well plate. The replication of the virus in supernatant was followed up by measuring p24 antigen on day 4. For virus control, it was performed by incubating the virus supernatant with HIV seronegative sera. To calculate percent neutralizing activity, p24 level of virus control was subtracted with p24 level of virus containing each dilution of MAb or sCD4 before being divided by p24 level of virus control and then multiplied by 100. The toxicity of MAb C2EB5 on PBMCs was assayed by adding various concentration of C2EB5 into 50,000 PBMCs (10^6 ^cells/ml) overnight. The viable PBMCs were counted after washing and stained with vital dye (typan blue).

### Determination of antigenic exposures on the surface of intact, native viruses

This procedure was modified from the protocol, which has been described previously [21,22]. Briefly, the flat-bottom 96-well plates were coated directly with 100 μl of each MAb or sCD4 (10 μg/ml carbonate buffer, pH 9.6). Following washing steps, the plates were blocked with blocking solution and incubated with 100 μl of virus supernatant (100 ng p24 antigen/ml) for 1 h. After washing steps to remove unbound viruses, 250 μl of 1% Triton X-100 was added to remove the contents of each well for measuring p24 antigen. The control well was performed by adding 100 μl of IL-2 medium and influenza virus (10 TCID50) instead of HIV-1 supernatant.

### Statistic analysis

All statistical analyses were performed on non-parametric analysis by program SPSS version 1.5. The association between antigenic exposures and HIV-1 subtypes were determined by Wilcoxon Signed-Rank test. The difference of antigenic epitope exposure of MAb C2EB5 and neutralizing activity was determined by Mann-Whitney U test.

## Results

### C1E1, C1E2 and C2E peptide immunogenicity

**Figure 1:**
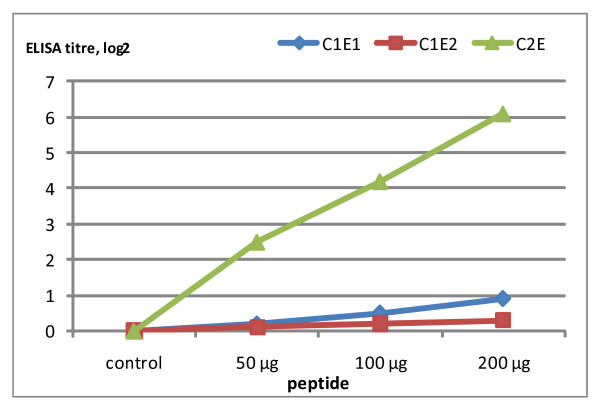
**Dose response curve of antibody production in immunized mice**. Sera from 5 mice per group were collected 1 week after the last immunization and tested by ELISA for the presence of specific antibodies. The peptides from C1E1 and C1E2 represented amino acids 93-112 of the C1 region, whereas peptides C2E represented amino acids 218-240 of the C2 region. Antibody titers of sera from mice immunized with C1E1, C1E2, and C2E at 50, 100, and 200 μg, are expressed as the log_2 _values of reciprocal endpoint titers. The control group was injected with normal saline. The immunization by peptides C1E1 and C1E2 induced a poor antibody response, whereas peptide C2E induced a robust antibody response, in a dose dependent manner.

Previously, we have investigated the activity of epitopes located at amino acids 93-112 of the C1 and 218-239 of the C2 regions as conserved neutralizable epitopes in HIV-1 CRF01_AE primary isolates by using peptides from C1E1, C1E2 and C2E to inhibit neutralizing activities of sera from HIV-1 CRF01_AE infected LTNPs [18]. The results led to the hypothesis that antibodies directed against theses epitopes should be broadly neutralizing antibodies. These peptides were used to immunize BALB/c mice. There were low titer antibody responses, as measured by peptide ELISA, in sera from mice immunized with peptides C1E1 and C1E2. However, peptide C2E induced antibody responses in BALB/c mice to a higher titer than that of the C1E1 and C1E2 peptides. The response to the C2E peptide occurred in a dose dependent manner (Figure 1). The mouse antisera from C2E immunization neutralized the HIV-1 CRF01_AE laboratory strain NP03 at a 1:30 dilution (data not shown). We therefore proceeded to produce monoclonal antibody from C2E immunized mice.

### Neutralizing activity of MAbs directed against peptide C2E

**Figure 2:**
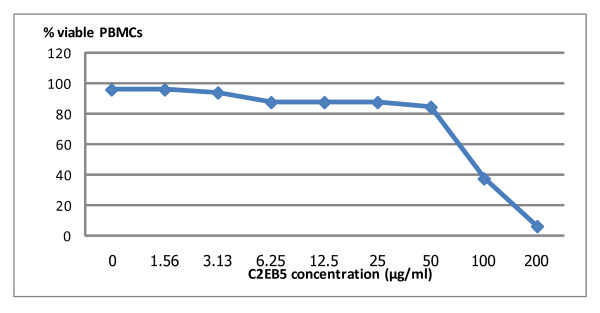
**Cellular toxicity of the C2EB5 MAb tested at various concentration on PBMCs**. Some toxic effects for the PBMC target cells were observed above concentrations > 50 ug/ml.

To explore the neutralizing activity of MAbs directed against peptide C2E, a murine MAb specific to this peptide was produced. The MAb clone B5 with the greatest neutralizing activity against NPO3 (CRF01_AE) HIV-1 strain was selected for further study and named MAb C2EB5. This MAb C2EB5 did not show any cross-reaction with peptides C1E1 and C1E2 by the ELISA method. The neutralizing activity of MAb C2EB5 was investigated against 14 isolates of HIV-1 from various subtypes including, subtype A; 92RW009 and VI191, subtype B; MN, IIIB, QH0692, and SF162, subtype C; 92BR025 and DU174, subtype D; 92UG024 and CRF01_AE; NPO3, CM244, NP1525, MENO23, MENO43. HIV-1 isolates used in this neutralization study were selected based on C2 amino acid similarity (CRF01_AE) and difference (other subtypes) to explore the cross-reactivity of the monoclonal antibody (Table 1). The results revealed that MAb C2EB5 neutralized subtype A, B, C, D and CRF01_AE with mean IC_50 _± SD 32.00 ± 6.92, > 50, 24.94 ± 21.11, 29.78 and 21.81 ± 6.71 μg/ml, respectively (Table 2). The cellular toxicity of MAb C2EB5 was demonstrated at a concentration > 50 μg/ml, as shown in Figure 2.

**Table 2:** Neutralization and virus capture (epitope exposure) of 14 HIV-1 isolates by monoclonal antibodies C2EB5, 4E10, 447-52D, and sCD4.

**HIV-1 strain**	**Subtype**	**IC**_50_**(μg/ml)**				**Virus binding activity**^3^ **(p24 antigen (pg/ml))**		
		
		**C2EB5**^1^	**447-52D**	**4E10**	**sCD4**	**C2EB5**	**447-52D**	**sCD4**
92RW009	A	36.89	22.5	17.5	8.7	11.33	7.26	6.8

VI191	A	27.1	15	13.75	8.7	11.84	17.78	7.33

QH0692	B	> 50	10.13	> 25	10	3.37	28.59	5.2

SF162	B	> 50	3.7	1.25	7.5	9.72	35.16	8.74

MN	B	> 50	6.25	13.75	9.89	6.25	20.6	5.4

IIIB	B	> 50	8.06	> 25	4.2	2.08	27.69	22.8

92BR025	C	10.01	15	9	10	38.16	5.08	29.44

DU174	C	19.87	1.5	1.25	10	33.13	24.19	32.74

92UG024	D	29.78	21.25	20	6.25	31.54	5.41	29.27

NPO3	AE	12.8	> 25	6.47	3.79	32.47	13.2	29.73

CM244	AE	17.5	21.5	7.5	10	35.55	15.17	28.71

NP1525	AE	12.7	> 25	15	1.87	30.1	2.51	18.68

MENO23^2^	AE	28.35	> 25	15	10	29.5	4.84	4.66

MENO43^2^	AE	27.72	> 25	17.3	10	21.62	0	11.23

^1^The neutralization activity of C2EB5 at concentrations more than 50 μg/ml was not reported because of cellular toxicity observed above this concentration. ^2^HIV-1 primary isolate ^3^The influenza virus was used as negative control virus in each test.

### The exposure of antigenic epitopes on the surface of intact, native HIV-1

The MAb C2EB5 was also characterized by performing virus capture to assess the exposure of the antigenic epitopes on the virion; data for C2EB5 was compared with capture data for the 4E10 and 447-52D MAbs and for sCD4. We initially assessed MAb binding to native, intact viruses by a virus binding ELISA [21]. We localized various epitopes by coating MAbs and sCD4 onto flatted-bottom 96-well plates, and adding the native viruses, without ionic detergent treatment, for attachment. The exposures of antigenic epitopes to these MAbs were compared. The virus binding activity (± SD) of HIV-1 subtypes A, B, C, D and CRF01_AE against MAb C2EB5 were 11.59 ± 0.36, 5.36 ± 3.39, 35.65 ± 3.56, 31.54 and 29.89 ± 5.18 pg/ml, respectively. HIV-1 subtype B virions showed the lowest amount of antibody capture by MAb C2EB5, and this was significantly lower than that observed for other subtypes (*p *< 0.05). This observation was correlated with low neutralizing activity of the C2EB5 MAb against HIV-1 subtype B isolates. HIV-1 subtype C demonstrated high antigenic epitope exposure to MAb C2EB5, which also correlated well with the neutralizing activity of the C2EB5 MAb against subtype C isolates. However, this comparison is based upon analysis of a small number of samples investigated in this study.

## Discussion

The antigenic diversity of HIV-1, particularly within the Env glycoprotein, is a major tool used by the virus as an immune evasion strategy, and this poses a major obstacle for the development of an effective HIV-1 vaccine. Therefore, a major focus of vaccine developers has been the difficulty in the elicitation of a broadly neutralizing antibody response. This effort has been directed towards a limited number of conserved epitopes on the envelope glycoprotein of HIV-1 primary isolates. Previously, we attempted to define these conserved neutralizable epitopes in CRF01_AE primary isolates from Thailand. We defined locations of various conserved epitopes and utilized these data to design synthetic peptides. We found that synthetic peptides representing amino acids 93-112 (C1E1 and C1E2) of C1 and 218-239 (C2E) of C2 regions could absorb NAbs in sera collected from Thai long-term non-progressors (LTNPs). Presence of these NAbs in sera of these subjects implies that these amino acids are associated with particular properties of neutralizable epitopes. Recently, these data were re-examined by creating a monoclonal antibody directed against peptide C2E to investigate its neutralization property.

Unfortunately, conserved neutralizable epitopes appear to be poorly immunogenic and Abs against them are rarely produced in infected subjects [23,24]. The peptides C1E1, C1E2 and C2E were also described to be poorly antigenic. Our previous study demonstrated that these peptides were bound at low titers by sera from HIV-1 infected individuals [18]. We found that peptide C2E could induce an antibody response in BALB/c mice whereas peptides C1E (C1E1 and C1E2) failed to do so. The differences amongst these peptides to induce antibody responses in BALB/c mice might be due to the fact that amino acid substitutions in peptide C2E results in the presence of highly immunogenic amino acids (His, Lys, Ala, Leu, Asp and Arg) within this epitope. These amino acids occur at a greater frequency than in the C1E peptides. The C2E (218-239) epitope is located around a β-turn near the loop α domain and C1E (93-112) spans the coil region located in the inner domain of gp120. These positions within C1E might be difficult for antibodies to recognize. However, previously, we found that there were NAbs against these epitopes in the sera of HIV-1 LTNPs [18]. The C1E and C2E epitopes might be less potent *in vitro *due to their lacking of conformational structure, combined epitopes, and allelic representations [25-27]. Indeed, the epitopes around amino acids 93-112 and 218-239 have been previously described, including epitopes at amino acids 90-100 of C1 and 222-231 of C2. Several MAbs against these epitopes have also been produced [23,24]. The MAb against 222-231 was reported to be reactive with a denatured form of gp120 [24], whereas we demonstrated that our MAb against amino acids 218-239 could neutralize native viruses albeit at high concentrations of MAb. This might be due to location of this epitope at the inner domain of gp120. While the MAb C2EB5 showed poor neutraliztion against the subtype B pseudovirus SF162 in the TZM-bl pseudovirus neutralization assay (data not shown) [28,29], it will be interesting to further test the breadth of this MAb, especially against subtype C isolates in this assay. A low IC50 against SF162 was also observed in the PBMC-based assay [IC50>50 μg/ml] (Table 2)

The reason that MAb C2EB5 was able to neutralize HIV-1 subtype C comparable to CRF01_AE may be due to the homology within the C2 amino acids (218-239) for these 2 subtypes (except at only one position at 227, Table 1). In contrast, HIV-1 subtype B contains 3-4 amino acid (position 218, 227, 231, 238) differences from CRF01_AE C2 amino acid. Interestingly, subtype A and D also have 3-4 amino acid (position 218, 227, 231, 237 or 238) differences from CRF01_AE, but they could be neutralized by MAb C2EB5 potently. The C2 (218-239) epitopes of subtypes A and D might be more exposed than subtype B epitopes because of shorter variable loops, such as V1-V4, or perhaps a lack of glycosylation sites that shield the conserved C2 neutralizable epitopes [30]. The neutralization resistance of HIV-1 subtype B against MAb C2EB5 was likely due to a reduced exposure of this epitope on the surface of this HIV-1 subtype B. However, this study is preliminary and further experiments will be required to confirm these observations.

## Conclusion

This is the first such study utilizing amino acid sequences of HIV-1 CRF01_AE primary isolates to design MAb. This MAb, in addition to neutralizing CRF01_AE, also cross-neutralizes other subtypes, particularly subtype C, which accounts for the largest population of HIV-1 infection in the world. As described above, high concentration of MAb C2EB5 was required to neutralize subtype B. However, it is our hope that MAbs directed against conserved regions are an alternative way to develop an effective vaccine against HIV. Accordingly, these data may facilitate our understanding of essential characteristics to design an immunogen to induce broadly neutralizing antibodies; this information may assist in the development of an effective HIV-1 vaccine.

## Abbreviations

CRF: circulating recombinant form; gp: glycoprotein; HRP: horse radish peroxidase; IFA: incomplete Freund's adjuvant; IL-2: interleukin-2; LTNP: long-term non-progressor; MAb: monoclonal antibody; NAb: neutralizing antibody; PBMC: peripheral blood mononuclear cell; PHA: phytohemagglutinin; sCD4: soluble CD4; SD: standard deviation; TCID_50_: 50% tissue culture infectious dose; TCLA: T-cell line adapted.

## Competing interests

The authors declare that they have no competing interests.

## Authors' contributions

AS participated in the design of the study, determined immunogenicities, performed MAb, investigated neutralizing activities, analyzed data and drafted the manuscript. JP participated in determining immunogenicities and performing MAb. WK and SS participated in the design of this study and were responsible for data analysis. NT prepared virus primary isolates and TCLA strains. RS conceived of the study, participated in the design of this study, analyzed data and drafted the manuscript. All authors have read and approved the final manuscript.
